# Effect of Oxidizing Agent on the Synthesis of ZnO Nanoparticles for Inverted Phosphorescent Organic Light-Emitting Devices without Multiple Interlayers

**DOI:** 10.3390/nano14070622

**Published:** 2024-04-02

**Authors:** Se-Jin Lim, Hyeon Kim, Hyun-A Hwang, Hee-Jin Park, Dae-Gyu Moon

**Affiliations:** Department of Electronic Materials, Device, and Equipment Engineering, Soonchunhyang University, Asan-si 31538, Republic of Korea; okis55@sch.ac.kr (S.-J.L.); rlagus910@sch.ac.kr (H.K.); hyuna366@sch.ac.kr (H.-A.H.); gomong6765@sch.ac.kr (H.-J.P.)

**Keywords:** OLED, inverted structure, ZnO nanoparticles, electron injection layer, oxidizing agent

## Abstract

Inverted organic light-emitting devices (OLEDs) have been aggressively developed because of their superiorities such as their high stability, low driving voltage, and low drop of brightness in display applications. The injection of electrons is a critical issue in inverted OLEDs because the ITO cathode has an overly high work function in injecting electrons into the emission layer from the cathode. We synthesized hexagonal wurtzite ZnO nanoparticles using different oxidizing agents for an efficient injection of electrons in the inverted OLEDs. Potassium hydroxide (KOH) and tetramethylammonium hydroxide pentahydrate (TMAH) were used as oxidizing agents for synthesizing ZnO nanoparticles. The band gap, surface defects, surface morphology, surface roughness, and electrical resistivity of the nanoparticles were investigated. The inverted devices with phosphorescent molecules were prepared using the synthesized nanoparticles. The inverted devices with ZnO nanoparticles using TMAH exhibited a lower driving voltage, lower leakage current, and higher maximum external quantum efficiency. The devices with TMAH-based ZnO nanoparticles exhibited the maximum external quantum efficiency of 19.1%.

## 1. Introduction

Organic light-emitting devices (OLEDs) are promising for large-area, high-resolution displays because of their excellent characteristics such as their high flexibility, thin thickness, and fast response [1]. The device performances have been significantly enhanced by introducing phosphorescent molecules, highly efficient fluorescent molecules, and advanced device architectures since the first report on efficient double-layer fluorescent OLEDs by S.A. VanSlyke and C.W. Tang in 1987 [2,3,4,5,6,7]. OLED-based displays are used for mobile phones, notebooks, and TVs due to their superiorities such as their visual quality, high contrast, and thin structure without a backlight unit [1,8]. New information technologies can be generated by OLED displays on flexible and stretchable substrates [9,10,11]. However, the device performances have been limited by the low electrical conductivity and low stability of the organic molecules [12,13,14,15].

ZnO nanoparticles are favorable in OLEDs because they have a high electron mobility, high chemical stability, and low conduction band minimum energy [16,17,18]. Since the ZnO nanoparticles are typically dispersed in organic solvents, solution processes are utilized to coat ZnO nanoparticle thin films. In conventional structures where the ZnO layer is formed on organic layers, the ZnO solution can degrade the underlying organic materials [19,20,21]. However, the organic layers are deposited on the nanoparticle layer in the inverted structures so that the organic layers are not damaged by the ZnO nanoparticle solutions. Therefore, the inverted structures are suitable for the ZnO nanoparticle devices [22,23,24,25,26]. ZnO nanoparticles are prepared on ITO cathodes in the inverted structures so that the surface roughness, electron mobility, and electrical conductivity of the ZnO nanoparticle layer can be improved by high-temperature annealing and surface treatment [23,24]. In addition, a high environmental stability can be achieved in the inverted devices since the air-stable ITO cathode is used instead of the highly reactive metal cathode in the inverted structures [22,23,24,25,26]. The inverted structure has advantages, for example, a low drop of luminance, low operating stress, and low voltage driving, in the active-matrix OLEDs where the drain line of the thin film transistor is directly connected to the cathode of the OLED [27,28].

Several ZnO-nanoparticle-based inverted devices have been reported using polymer or phosphorescent emitters [29,30,31,32]. The inverted devices with poly(9-dioctylfluorene-alt-benzothiadiazole) were demonstrated by Lu et al. [29]. The current efficiency was 27.6 cd/A. Super-Yellow-based inverted polymer devices were reported by Höfle et al. [30]. The efficient phosphorescent inverted devices were also demonstrated by Zhang et al. [31]. They achieved a current efficiency of 60.9 cd/A. Zhou et al. reported the inverted green phosphorescent devices with a quantum efficiency of 21.2% [32]. They used the additional electron-injecting interlayers such as Ba(OH)_2_, a thin Ag nanoparticle layer, and polyethyleneimine on the ZnO layer [29,30,31,32,33]. Although these interlayers enhance the injection of electrons into the emission layer, these methods make the device fabrication process more complicated.

In this work, we demonstrate the inverted phosphorescent devices without additional interlayers between the ZnO electron injection layer and the organic electron transport layer. Few authors reported the inverted phosphorescent devices using ZnO nanoparticles without additional interlayers [34,35,36]. Lee et al. demonstrated blue phosphorescent devices with the ZnO nanoparticle layer [34]. The maximum external quantum efficiency (EQE) was 8.2% in their inverted devices. Hwang et al. demonstrated the ZnO-nanoparticle-based inverted phosphorescent devices with an EQE of 15.8% [35]. Recently, Li et al. reported on the inverted phosphorescent devices using a palladium(II)-based phosphorescent aggregate emitter [36]. They achieved an EQE of 23.9% in their devices. In this work, we synthesized ZnO nanoparticles using different oxidizing agents of tetramethylammonium hydroxide pentahydrate (TMAH) and potassium hydroxide (KOH) for the inverted phosphorescent devices. Various oxidizing agents have been used for synthesizing ZnO nanoparticles in preparing the inverted devices [23,36]. For example, Pu et al. used KOH as an oxidizing agent for the spherical, rodlike, and intermediate-type ZnO nanoparticles [23]. They fabricated the conventional and inverted OLEDs using KOH-based nanoparticles. On the other hand, Li et al. utilized a TMAH oxidizing agent for synthesizing ZnO nanoparticles with an average particle size of 3.0 nm [36]. However, there is no report on the effect of oxidizing agents in achieving highly efficient inverted OLEDs. In this paper, we used KOH and TMAH as oxidizing agents. KOH has been widely used for synthesizing ZnO nanoparticles, while TMAH has been sometimes used to synthesize ZnO nanoparticles in the OLEDs. Therefore, the comparison of these two oxidizing agents can be a good guide for the ZnO nanoparticles of OLEDs. In addition, the band gap, surface roughness, and electrical resistivity of ZnO nanoparticles strongly depend on the oxidizing agents. The inverted devices with TMAH-based ZnO nanoparticles exhibited a lower driving voltage, lower leakage current, and higher maximum EQE compared to the KOH-based devices. The maximum EQE of 19.14% was achieved in the TMAH-based devices.

## 2. Materials and Methods

### 2.1. Preparation of ZnO Nanoparticles

ZnO nanoparticles were synthesized using different oxidizing agents of TMAH and KOH. TMAH was used for synthesizing ZnO nanoparticles as follows: 15 mL dimethyl sulfoxide (DMSO), 0.75 mmol zinc acetate dihydrate, 1.16 mmol TMAH, and 5 mL ethanol were used for synthesizing ZnO nanoparticles. DMSO and ethanol were used for dissolving zinc acetate dihydrate and TMAH, respectively. After mixing zinc acetate dihydrate solution and TMAH solution, ethyl acetate was added to precipitate ZnO nanoparticles. The collected ZnO nanoparticles by centrifugation were dispersed in ethanol. KOH was used for synthesizing ZnO nanoparticles as follows: 16.8 mL ethanol, 0.1 mL deionized water, and 1.83 mmol zinc acetate were used to prepare zinc precursor solution. The zinc acetate was completely dissolved by heating the mixed solution at 60 °C. Then, 3.49 mmol of KOH was dissolved in 9.2 mL of methanol. The KOH solution was mixed with the zinc acetate solution. The methanol solvent in mixed solution was evaporated at 60 °C for 30 min, followed by stirring the concentrated mixture at 60 °C for 6 h. The supernatant was carefully removed after washing the mixed solution with 20 mL of methanol. After repeating this methanol washing process three times, the collected ZnO nanoparticles were dispersed in the mixed solution of chloroform and 2-ethoxyethanol.

### 2.2. Device Fabrication

ITO-coated glass substrate with a sheet resistance of about 10 Ω/square was used for fabricating the inverted phosphorescent OLEDs. A standard photolithography process was used to define the ITO cathode patterns. The patterned ITO substrate was ultrasonically cleaned with acetone, acetone, isopropyl alcohol, and deionized water. The organic residues on the cleaned substrate were removed by exposing to oxygen plasma. Then, 10 nm-thick ZnO nanoparticle layer was spin-coated to deposit the electron injection layer on the ITO cathode substrate. The spin-coating speed and time were 2000 rpm and 15 s, respectively. Vacuum thermal evaporation method was used to deposit the organic and metal layers. The base pressure was kept at 1 × 10^−6^ Torr during evaporation. For the electron injection layer, 2,2′,2″-(1,3,5-benzinetriyl)-tris-(1-phenyl-1-H-benzimidazole) (TPBi) was evaporated. The thickness of TPBi layer was 25 nm. Tris(2-phenylpyridine)iridium(III) [Ir(ppy)_3_] and 4,4′-bis(9-carbazolyl)biphenyl (CBP) were used as phosphorescent dopant and host molecules, respectively. Then, 5 wt% Ir(ppy)_3_ and CBP were co-evaporated to deposit 20 nm-thick emission layer. Undoped CBP was evaporated to deposit hole transport layer with a thickness of 20 nm. MoO_3_ was evaporated for 10 nm-thick hole injection layer. After this, Al was evaporated to define 100 nm-thick anode. The inverted phosphorescent device of ITO/ZnO/TPBi/CBP:Ir(ppy)_3_/CBP/MoO_3_/Al was completed by encapsulating in nitrogen atmosphere.

### 2.3. Characterizations

The crystal structure of ZnO nanoparticles was investigated by X-ray diffraction (Miniflex 600, Rigaku, Tokyo, Japan) with Cu-Kα radiation of 0.15406 nm. The thickness of the thin film including ZnO nanoparticle layer was measured using a surface profilometer (Surfcorder, ET3000, Kosaka Laboratory Ltd., Tokyo, Japan). JEM-ARM200F (JEOL Ltd., Tokyo, Japan) transmission electron microscopy (TEM) instrument was used to investigate the shape and particle size of the ZnO nanoparticles. Scanning electron microscopy (SEM) using a XL-30 ESEM with EDAX (Philips, Amsterdam, Netherlands) was used to obtain surface morphology of nanoparticles. XE7 (Park Systems, Suwon, Republic of Korea) atomic force microscopy (AFM) was utilized to measure the surface roughness. Ultraviolet–visible (UV–vis) absorption was measured by Lamda 35 spectrometer (PerkinElmer, Waltham, MA, USA). Keithley 2400 (Keithley, Solon, OH, USA), calibrated Si photodiode, and CS1000 spectroradiometer (Konica Minolta, Tokyo, Japan) were used to measure the voltage, current density, and luminance of ZnO nanoparticle films and devices. CS1000 spectroradiometer was used to measure the photoluminescence and electroluminescence spectra of the nanoparticles and devices.

## 3. Results and Discussion

**Figure 1:**
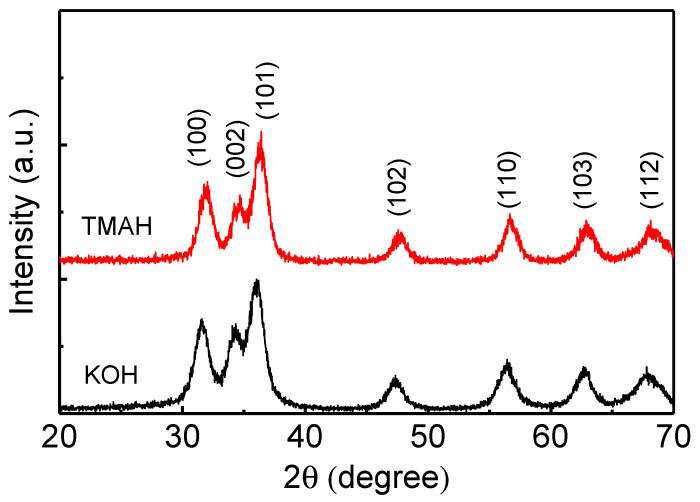
X-ray diffraction patterns for the ZnO nanoparticles synthesized using TMAH and KOH.

ZnO nanoparticles were prepared using different oxidizing agents by the solution–precipitation method. TMAH and KOH were used as oxidizing agents. The X-ray diffraction patterns shown in Figure 1 were measured to investigate the crystal structure of the ZnO nanoparticles synthesized using TMAH and KOH. ZnO nanoparticle powder was used for the analysis of the X-ray diffraction. ZnO nanoparticles exhibit diffraction peaks at 31.5°, 34.2°, 36.1°, 47.7°, 56.5°, 62.8°, and 68.0°, which peaks correspond to (100), (002), (101), (102), (110), (103), and (112) planes, respectively. This result indicates that all the ZnO nanoparticles using KOH and TMAH have a hexagonal wurtzite structure (JCPDS 36-1451) [35].

**Figure 2:**
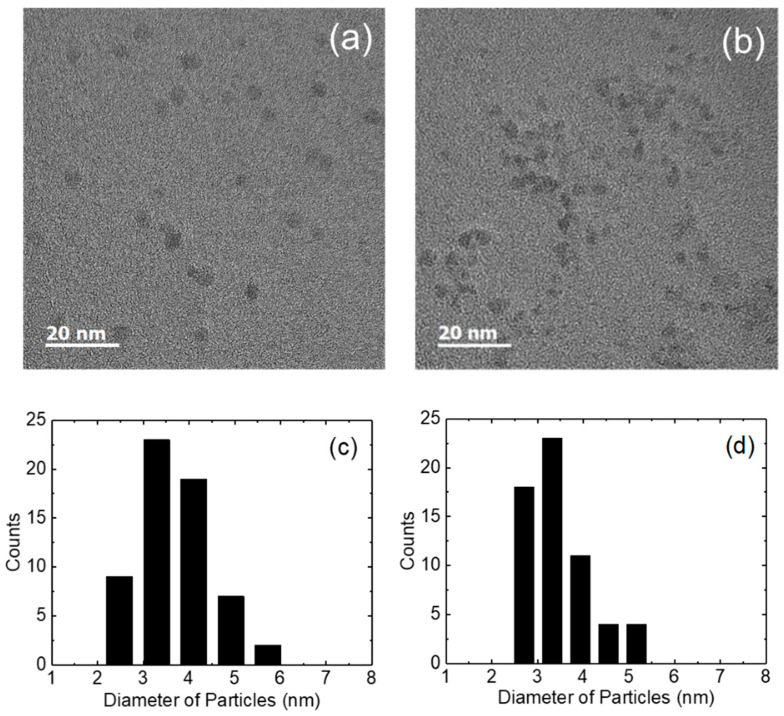
Transmission electron microscopy images for the (**a**) TMAH- and (**b**) KOH-based ZnO nanoparticles, and particle counts as a function of particle diameter for the (**c**) TMAH- and (**d**) KOH-based ZnO nanoparticles.

Figure 2a,b show the TEM images of ZnO nanoparticles using TMAH and KOH. TMAH-based nanoparticles were almost spherical in shape. The average particle size of TMAH-based nanoparticles was 4.1 nm. On the other hand, KOH-based ZnO nanoparticles show that the oval and spherical particles are mixed. The average particle diameter of 3.8 nm was measured in the KOH-based nanoparticles. Figure 2c,d show the particle size distributions of the TMAH- and KOH-based nanoparticles. Smaller particles are distributed in the KOH-based nanoparticles. It has been reported that the shape of the KOH-based nanoparticles is sensitive to the concentration of the zinc precursor and oxidizing agent [23,26,37]. The oval-shaped nanoparticles may have resulted from the concentrated solution of zinc acetate and the oxidizing agent by the evaporation of methanol at 60 °C during the synthesis process.

**Figure 3:**
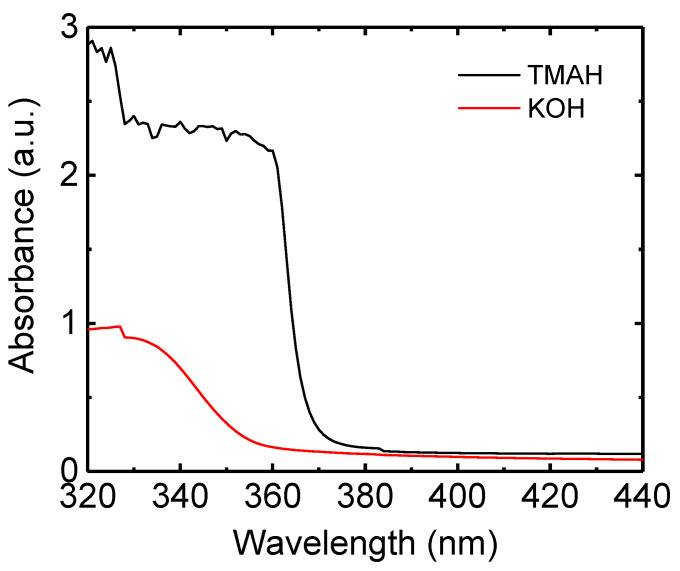
Absorption spectra for the ZnO nanoparticles synthesized using TMAH and KOH.

Figure 3 shows the absorption spectra for the ZnO nanoparticles synthesized using TMAH and KOH. ZnO nanoparticle solutions were used for the measurement of the absorption spectra. The absorption spectrum of the TMAH-based nanoparticles is red-shifted compared with the KOH-based nanoparticles, indicating that the TMAH-based ZnO nanoparticles have a smaller band gap than the KOH-based nanoparticles. The band gap energy of ZnO nanoparticles was measured from the extrapolation of the absorption edge and the wavelength axis [16]. The measured band gaps are 3.37 and 3.46 eV for the TMAH- and KOH-based nanoparticles, respectively. It has been known that the band gap of bulk ZnO is 3.2–3.3 eV [38]. Therefore, the higher band gaps in the synthesized nanoparticles indicate the spatial confinement of the photogenerated carriers. The effective mass approximation or tight binding model has been applied to estimate the band gap of the quantum-confined particles which depends on the particle size [39,40]. The band gap was estimated using the effective mass approximation model and tight binding model. The estimated band gaps using effective mass approximation were 3.72 and 3.81 eV for the TMAH (4.1 nm)- and KOH (3.8 nm)-based ZnO nanoparticles, respectively. Hence, the difference between the two particles was 0.9 eV. However, the effective mass approximation model overestimates the measured band gaps since the measured band gaps were 3.37 and 3.46 eV for the TMAH- and KOH-based nanoparticles, respectively. On the other hand, the band gaps using the tight binding model were 3.41 and 3.44 eV, respectively, for the TMAH- and KOH-based nanoparticles. The tight binding model results in more accurate results of the band gap.

**Figure 4:**
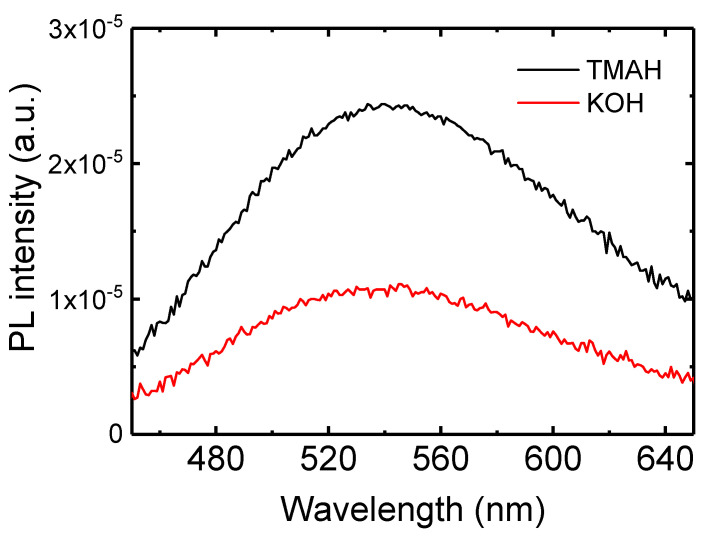
Photoluminescence spectra for the ZnO nanoparticles synthesized using TMAH and KOH.

Photoluminescence (PL) spectra of the ZnO nanoparticles synthesized using TMAH and KOH were measured (Figure 4). ZnO nanoparticle solutions were used to measure the PL spectra. The broad emission can be observed in the visible range, resulting from surface defects of ZnO nanoparticles [16,41]. The visible emission peaks can be observed at 545 and 535 nm for the TMAH- and KOH-based nanoparticles, respectively. The PL peak is related to the defect level and the band gap of nanoparticles [16]. The larger particle size in the TMAH-based nanoparticles results in the narrower band gap compared with the KOH-based nanoparticles. The spectra also indicate the lower PL intensity in the KOH-based nanoparticles. It has been known that the PL intensity in the visible range depends on the amount of surface defects and the crystallinity in ZnO nanoparticles [16,26,41]. Therefore, the lower PL intensity in the KOH-based nanoparticles suggests a lower amount of surface defects and the higher crystallinity.

**Figure 5:**
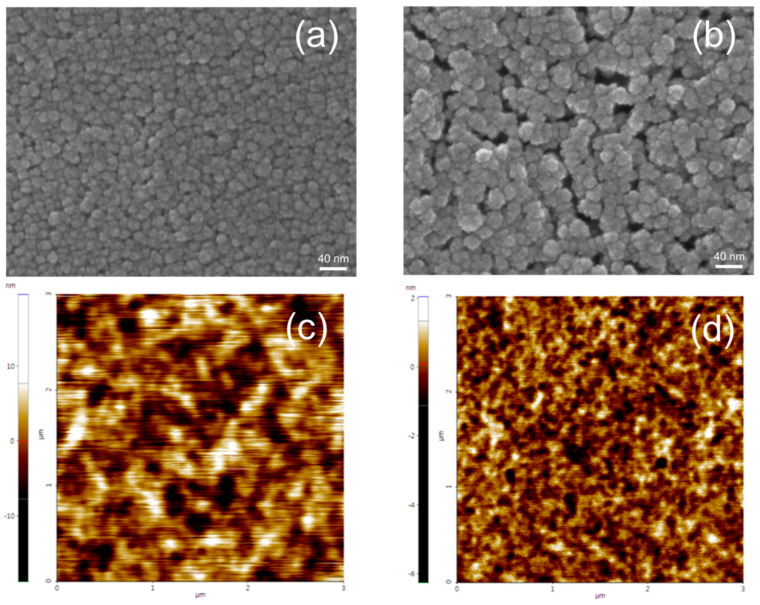
SEM (**upper**) and AFM (**lower**) images for the (**a**,**c**) TMAH- and (**b**,**d**) KOH-based ZnO nanoparticle films.

ZnO nanoparticles using TMAH and KOH were spin-coated to investigate the uniformity and surface morphology of the coated film. The surface of the ZnO nanoparticles synthesized using different oxidizing agents was investigated by SEM and AFM (Figure 5). Then, 40 nm-thick ZnO nanoparticle films were used for the SEM and AFM images. The SEM images show that the particle size is about 10–30 nm, which is larger than the TEM particle size of Figure 2. However, the close observation of SEM images reveals that the observed particles consist of few nanometer-size particles, indicating the agglomeration of nanoparticles. The SEM images indicate that severe agglomeration occurs in the KOH-based film, while the TMAH-based ZnO nanoparticle film exhibits a uniform and continuous surface. The aggregation of particles in the KOH-based film can be observed in the previous report [42]. This aggregation of nanoparticles results in a rough surface. Figure 5b,c show the AFM images for the ZnO nanoparticles using TMAH and KOH. AFM images are consistent with the SEM images. A serious agglomeration of nanoparticles can be observed in the KOH-based nanoparticle films. The KOH-based film exhibited the surface roughness of 3.1 nm, while a much lower value of 0.41 nm was obtained in the TMAH-based film. The rough surface in the KOH-based film results from the agglomerated nanoparticles. Since the several nanoparticles are severely merged into the agglomerated particles in the KOH-based film, the agglomerated regions and vacant regions are distinguished, resulting in a rough surface.

**Figure 6:**
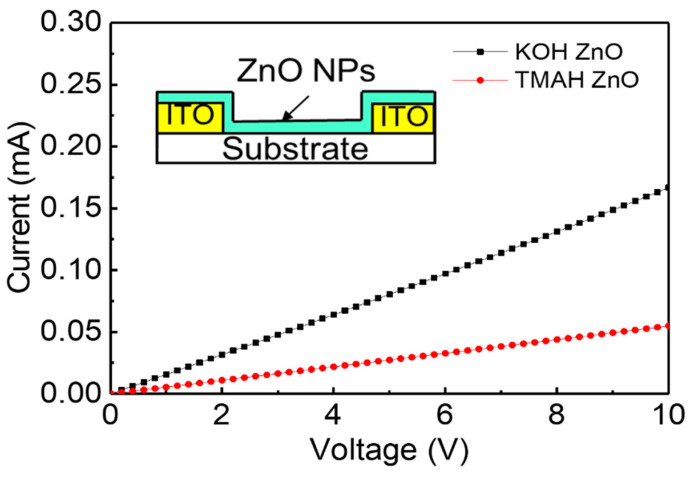
Current–voltage curves for the thin film resistors of the ZnO nanoparticles synthesized using TMAH and KOH. Inset: structure of ZnO nanoparticle thin film resistor.

Thin film resistors were fabricated to investigate the electrical conduction characteristics of the synthesized nanoparticles. The inset of Figure 6 shows the structure of the ZnO nanoparticle film on the ITO patterned substrate. The thickness, length, and width of the resistor were 80 nm, 10 μm, and 4 cm, respectively. Figure 6 shows the electrical conduction characteristics of the TMAH- and KOH-based ZnO nanoparticle films. The current increases linearly with the voltage, indicating that the ZnO nanoparticle film forms an ohmic contact with ITO electrode. The current at the same voltage is higher in the KOH-based ZnO nanoparticle film. For example, the KOH-based film exhibits a current of 0.08 mA at 5 V. On the other hand, 0.03 mA is measured at the same voltage in the TMAH-based film. The electrical resistivity was measured from the current–voltage curve. The electrical resistivities were 5.8 × 10^3^ and 2.3 × 10^3^ Ωcm in the TMAH- and KOH-based films, respectively. It has been reported that the electrical conduction in the ZnO nanoparticle film depends on the surface defects of the nanoparticles [43]. The lower electrical resistivity in the KOH-based film may be due to the lower defect states as shown in the PL spectra of Figure 4.

**Figure 7:**
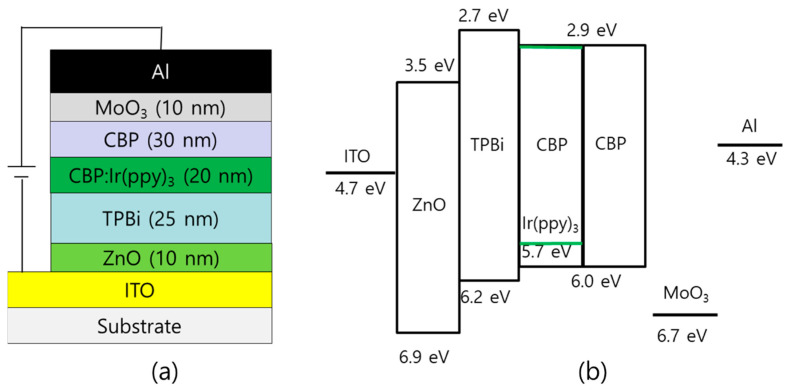
(**a**) The structure of the inverted device and (**b**) corresponding band diagram of the device.

Inverted phosphorescent OLEDs were fabricated using the ZnO nanoparticles synthesized with different oxidizing agents of TMAH and KOH. The structure of the inverted device was ITO/ZnO/TPBi/CBP:Ir(ppy)_3_/CBP/MoO_3_/Al. Figure 7 shows the structure of the inverted device and corresponding band diagram. In our devices, the additional interlayer between the ZnO nanoparticles and the TPBi was not used because the efficient injection of electrons into the TPBi layer can be possible through the ZnO layer in this structure [35].

**Figure 8:**
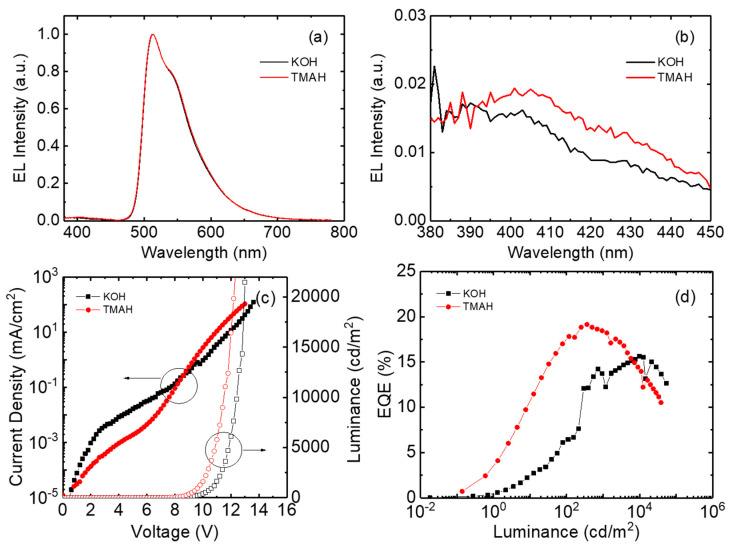
Electroluminescence spectra (**a**) in the visible and (**b**) blue wavelength region, and (**c**) current–voltage–luminance and (**d**) EQE curves for the inverted OLED TMAH- and KOH-based inverted OLEDs.

Figure 8a,b show the electroluminescence spectra of the inverted devices with ZnO nanoparticles synthesized using TMAH and KOH. The emission spectrum exhibits a strong intensity at 514 nm, owing to the phosphorescent emission from the Ir(ppy)_3_ molecules [44]. In addition, the weak emission from the CBP hole transport layer can be observed at around 400 nm. This parasitic radiative recombination indicates that the electrons are leaked into the CBP hole transport layer. The parasitic emission also suggests that the recombination takes place in the emission layer adjacent to the CBP hole transport layer. The parasitic emission intensity is slightly higher in the TMAH-based device, indicating a higher electron injection in the TMAH-based device.

Figure 8c,d show the current density–voltage–luminance and EQE curves for the TMAH- and KOH-based inverted devices. The KOH-based device exhibits a higher leakage current, attributed to the rough surface resulting from the agglomeration of nanoparticles (Figure 5). It should be noted that the SEM and AFM images of Figure 5 were obtained using 40 nm-thick ZnO nanoparticle films. On the other hand, a 10 nm-thick ZnO nanoparticle layer was used for the inverted devices. The larger thickness may not reflect the real situation within the OLED. However, from the AFM measurement, ZnO nanoparticle films synthesized using KOH under several conditions (for example, a different DI water ratio) were measured to be always rough compared to the TMAH-based ZnO films. The surface roughness of the KOH-based films was over 2–3 nm, but the TMAH films exhibited a surface roughness less than 1 nm for all the measured samples. Therefore, we can postulate that the surface of the KOH-based film is rough compared to the TMAH-based films even though both the KOH- and TMAH-based films have a 10 nm thickness in the devices. A lower driving voltage can be observed in the TMAH-based device. The driving voltages for obtaining 10 mA/cm^2^ are 11.0 and 11.8 V in the TMAH- and KOH-based devices, respectively. The turn-on voltage that is defined as a voltage for 1 cd/m^2^ is also lower in the TMAH-based device. The turn-on voltages are 6.6 and 7.0 V in the TMAH- and KOH-based devices, respectively. The TMAH-based device exhibits a higher luminance at a lower voltage. For example, 9.6 V is required to achieve 1000 cd/m^2^ in the TMAH-based device. However, the KOH-based device exhibits 10.6 V at 1000 cd/m^2^. Since the thickness of the ZnO layer is 10 nm, the ZnO nanoparticle layer contributes to the electron injection rather than the electron transport. Therefore, the lower driving voltage suggests that the electron injection is higher in the TMAH-based device. The higher efficiency of the electron injection in the TMAH-based devices can be confirmed by the higher parasitic radiative recombination of Figure 8b. The better electron injection in the TMAH-based device may result from the smooth and continuous surface in the TMAH-based ZnO layer. The rough and porous surface may result in poor contact with the upper organic electron transport layer, suppressing the injection of electrons. The charge balance is typically limited by the injection of electrons in the inverted OLEDs with ZnO nanoparticles [29,30,31,32,33,34,35,36]. Therefore, the better electron injection in the TMAH-based device results in the higher recombination efficiency. Figure 8d shows the EQE curves for the TMAH- and KOH-based devices. The maximum EQE is higher in the TMAH-based device. The KOH-based device exhibits an EQE of 15.6%. On the other hand, the TMAH-based device shows a higher maximum EQE of 19.1%, indicating a better electron–hole balance due to the efficient injection of electrons in the inverted device.

## 4. Conclusions

Hexagonal wurtzite ZnO nanoparticles were synthesized using different oxidizing agents of TMAH and KOH. The average particle sizes were 4.1 and 3.8 nm in the TMAH- and KOH-based nanoparticles, respectively. The widened band gaps due to the spatial confinement of the charge carriers were 3.37 and 3.46 eV in the TMAH- and KOH-based nanoparticles, respectively. The electrical resistivity of the TMAH-based nanoparticles was 5.8 × 10^3^ Ωcm, while the lower surface defects resulted in a lower electrical resistivity of 2.0 × 10^3^ Ωcm in the KOH-based nanoparticles. The high leakage current in the inverted OLEDs with KOH-based nanoparticles was attributed to the rough surface resulting from the agglomeration of the nanoparticles. The inverted phosphorescent device with TMAH-based nanoparticles exhibited a higher maximum EQE of 19.1% and a lower driving voltage compared to the KOH-based device. These results suggest that the highly efficient inverted OLEDs are possible without an additional interlayer. We expect that this comparison of oxidizing agents can be a good guide in synthesizing ZnO nanoparticles for the inverted devices, and TMAH-based ZnO nanoparticles can be an excellent candidate for the electron injection layer of the highly efficient inverted devices.

## Data Availability

Data are contained within the article.
